# Utility of Skeletal Muscle CT in Diagnosing Spinal Muscular Atrophy Type 3 in a Patient Who Had Been Undiagnosed for 50 Years

**DOI:** 10.7759/cureus.38709

**Published:** 2023-05-08

**Authors:** Michihiro Hirayama, Takashi Ayaki, Daisuke Yoshii, Ken Yasuda, Ryosuke Takahashi

**Affiliations:** 1 Neurology, Kyoto University Graduate School of Medicine, Kyoto, JPN

**Keywords:** smn1 gene, diagnosis delay, sma type 3, fatty infiltration, muscle atrophy, skeletal computed tomography, spinal muscular atrophy (sma)

## Abstract

A 69-year-old woman presented with progressive limb weakness lasting 50 years. She denied any congenital disorders or a family history of neuromuscular disease. At ages 29, 46, and 58 years, she underwent hospitalization and evaluations including electromyogram (EMG) and muscle biopsy, but the results were inconclusive. As a result, she received a tentative diagnosis of myopathy of unknown etiology. However, at the age of 69 years, a computed tomography (CT) scan of her skeletal muscles revealed severe involvement of the triceps brachii, iliopsoas, and gastrocnemius muscles, along with preservation of the biceps brachii, gluteus maximus, and tibialis anterior muscles, which was consistent with spinal muscular atrophy (SMA). Finally, genetic testing revealed the deletion of the survival of the motor neuron 1 (*SMN1*) gene, confirming the diagnosis of SMA type 3. As our case suggests, SMA patients with prolonged disease duration could be underdiagnosed even after EMG and muscle biopsy. A skeletal CT scan could be useful for the diagnosis of SMA patients compared with MRI.

## Introduction

Spinal muscular atrophy (SMA) is an autosomal recessive motor neuron disease characterized by degeneration of the anterior horn of the spinal cord. The most common causative gene of SMA is the survival of the motor neuron 1 (*SMN1*) gene on chromosome 5, and a definitive diagnosis of SMA requires genetic analysis [1].

Currently, several therapeutic options are available [2,3], and research has shown that the shorter the duration between symptom onset and administration, the better the outcome [2]. Therefore, an early diagnosis is becoming increasingly important. However, distinguishing adult SMA from other neuromuscular diseases can sometimes be difficult [4].

Several imaging studies have reported specific muscle involvement patterns in SMA types 2 and 3 [5,6]. We report a case that was misdiagnosed as having a myopathy for a long time but whose selective involvement pattern in the skeletal muscle CT led to reconsideration and a genetic diagnosis of SMA.

## Case presentation

A 69-year-old Japanese woman presented with progressive weakness. Her medical history was unremarkable, except for urinary stones and fatty liver. She denied any family history of neuromuscular disease, and her parents were non-consanguineous. She quit smoking in her 20s, and she denied drinking alcohol. She had no abnormalities at birth or in motor development until she was a toddler. She was short in stature and inept at sports during her school years.

At the age of 19 years, the patient frequently noticed muscle cramping in her legs. Sometimes she tripped on stairs and could not walk as fast as others. She first visited our hospital at the age of 29 years. Electromyography (EMG) of the legs revealed chronic denervation and a muscle biopsy of the left rectus femoris was inconclusive.

**Figure 1:**
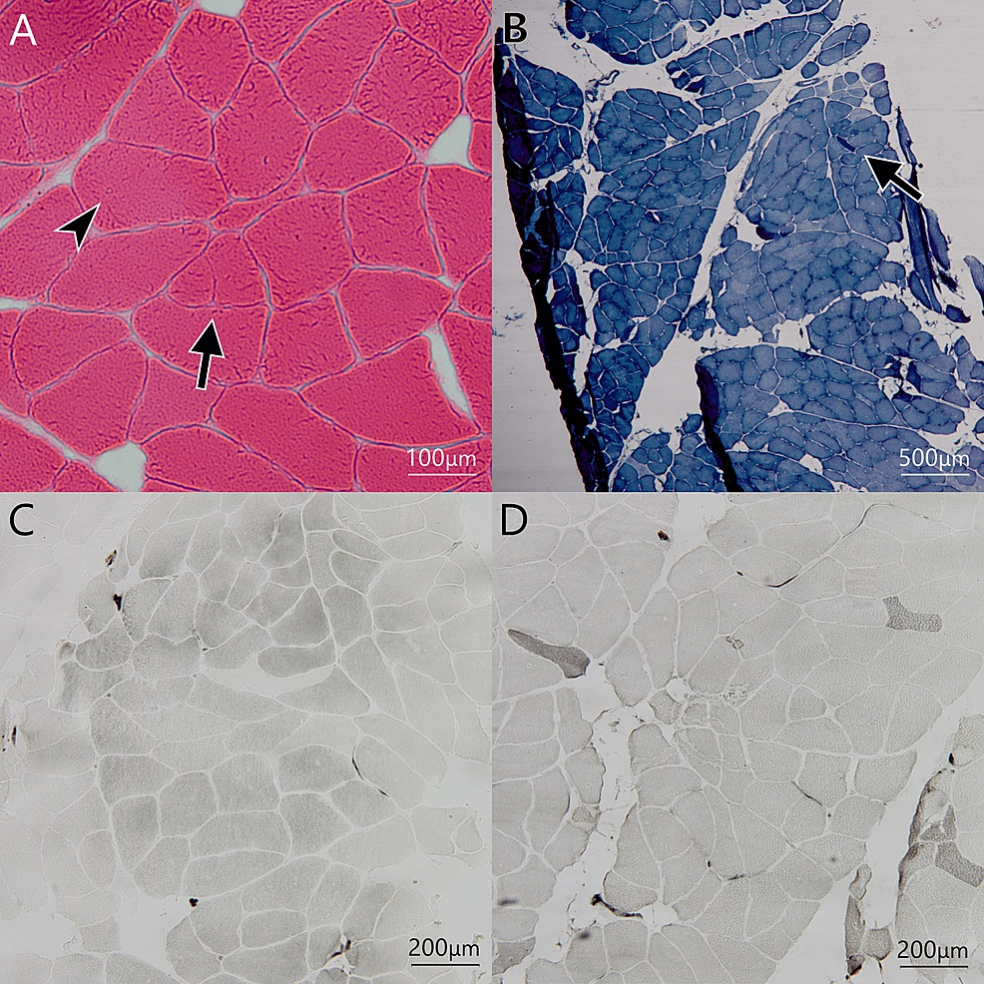
Muscle biopsy from left brachioradialis at the age of 46 years (A) Hematoxylin and eosin (HE) staining. The central nuclei (arrowhead) and splitting fibers (arrow) were observed. Connective tissue was slightly increased. Large group atrophy fibers were not observed. (B) Nicotinamide adenine dinucleotide dehydrogenase‐tetrazolium reductase (NADH-TR) staining. The neurogenic change was not observed except for a few small angular fibers (arrow). (C, D) Adenosine triphosphatase (ATPase) staining (pH 4.5 and 10.6). Type 1 fiber predominance was observed and type 2B fiber was rarely observed. Type 2C fiber was not seen.

Her symptoms gradually increased in severity, and she required a cane to walk at the age of 39 years. At the age of 46 years, she was hospitalized for the second time. Because the creatinine kinase (CK) level was at the upper limit of the normal range or slightly elevated and muscle specimens from the left brachioradialis muscle showed a predominance of type 1 fiber (Figures 1A-1D), she was tentatively diagnosed with myopathy of an unknown etiology. She had not been prescribed any medication such as glucocorticoids but had been followed up in the outpatient clinic.

**Figure 2:**
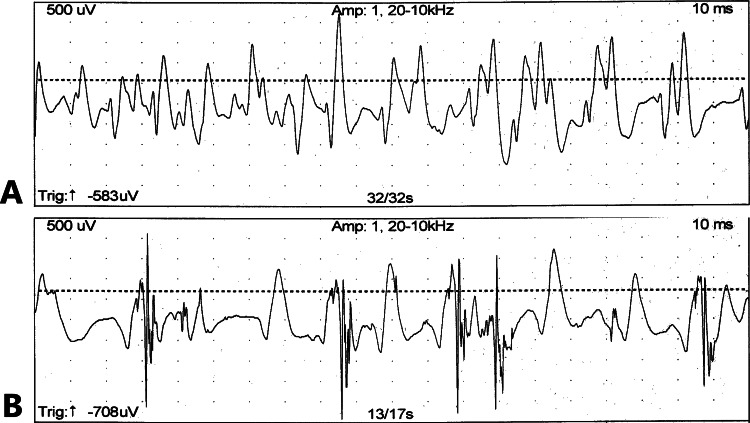
Needle electromyography (EMG) at the age of 58 years (A) EMG at the right deltoid revealed an early recruitment pattern. (B) EMG at the right biceps brachii showed a late recruitment pattern and polyphasic motor unit potential.

The patient became unable to walk and required a wheelchair in her 50s. At the age of 58 years, she was admitted to the hospital for the third time. She was 142 cm tall and weighed 53.7 kg (with a body mass index (BMI) of 26.6 kg/m^2^). EMG showed a normal to mildly early recruitment pattern in the right deltoid, biceps brachii, first dorsal interosseous, and iliopsoas muscles. However, the late recruitment pattern and increased amplitude were also seen in the deltoid and biceps brachii (Figures 2A, 2B), and a muscle biopsy of the right rectus femoris muscle did not yield sufficient tissue due to severe muscle atrophy.

At the age of 69 years, the patient weighed 54.6 kg (BMI 27.1 kg/m^2^) and did not display any cognitive impairment, executive dysfunction, cranial nerve abnormalities including tongue atrophy or fasciculation, dysphagia, dysarthria, fasciculations, or sensory impairment. Muscle atrophy of the limb was not evident due to obesity.

The patient’s muscle strength score, determined using the manual muscle test (MMT), was 4-5 in her neck flexor and extensor, deltoid, and biceps brachii muscles, while the MMT scores reduced to 2-3 in her triceps brachii and all lower limb muscles. The patient’s grip strength was 5 and 3 kg on the right and left sides, respectively. Her muscle tonus was hypotonic in all limbs. Her upper limbs showed postural tremors. Deep tendon reflexes decreased in all muscles except the biceps brachii and brachioradialis tendon, and her Babinski reflex was bilaterally absent.

We observed no abnormalities in the patient’s complete blood count or blood chemistry tests. The CK levels were between the upper range and twice the normal limit (about 120-300 mg/dL) for 20 years. Results of the brain and whole spine MRI scans were unremarkable. The peripheral nerve conduction study did not detect any abnormalities except for mild carpal tunnel syndrome in the right hand and a decrease in amplitude in the right tibial compound muscle action potential, which was less than 50% of the normal lower limit.

**Figure 3:**
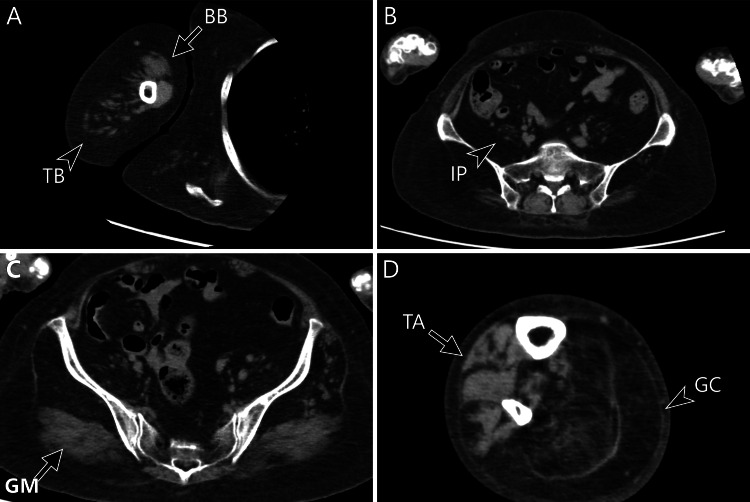
Computed tomography image of the extremities at the age of 79 years (A) The right triceps brachii (TB: arrowhead) was more severely impaired than the biceps brachii (BB: arrow). (B, C) The iliopsoas (IP: arrowhead) was more severely impaired than the gluteus maximus (GM: arrow). (D) Similarly, the right gastrocnemius (GC: arrowhead) was more severely impaired than the tibialis anterior (TA: arrow).

A muscle CT scan of the patient’s extremities showed fat replacement in the triceps brachii, iliopsoas, and gastrocnemius muscles. In contrast, the biceps brachii, gluteus maximus, and tibialis anterior were relatively preserved (Figures 3A-3D); a muscle ultrasound was not performed. Genetic testing using a multiplex ligation dependent probe amplification assay revealed the deletion of *SMN1* and four copies of *SMN2*. Based on the above results, she was diagnosed as SMA type 3.

After the diagnosis, she refused nusinersen injection to avoid hospitalization. She chose to be treated with risdiplam. Her post-treatment progress was monitored with clinical information only. She maintained muscle strength and experienced no adverse events.

## Discussion

We have successfully diagnosed a patient with SMA type 3, which had remained undiagnosed for 50 years, using skeletal muscle CT. Diagnosis of adult SMA is sometimes difficult, and some previous reports highlighted the long delay from symptom onset to genetic diagnosis [4]. In a cohort study in Italy, the mean time between symptom onset and diagnosis was 5.28 months for type 2 SMA and 16.8 months for type 3 SMA [7]. In this patient, the duration was 50 years, which is much longer than in previous reports.

Delays in diagnosis are thought to occur for the following reasons. First, SMA patients can have various clinical presentations, ranging from the severe infantile type to the milder adult-onset type. This makes it difficult for clinicians to get a clinical image. Second, SMA can present clinical features similar not only to motor neuron diseases but also to peripheral nerve disorders and even myopathy [8,9]. Third, especially in patients with a long disease course, EMG or muscle biopsy does not show typical neurogenic changes [10]. In our case, the patient presented with more severe muscle atrophy in the proximal muscles than in the distal muscles, and CK levels were mildly elevated. In addition, the EMG showed both early and late recruitment. The muscle biopsy showed type 1 fiber predominance, which is often seen in muscular dystrophy or congenital myopathy. These findings are atypical for a motor neuron disease and led to a misdiagnosis as myopathy.

Previously, several studies have shown that selective muscle involvement patterns are seen not only in myopathies but also in motor neuron diseases, and it could provide clues to clarify the diagnosis [11]. Amyotrophic lateral sclerosis often shows atrophic first dorsal interosseous and preserved abductor digit minimi, known as the “split hand syndrome” [12]. Spinal and bulbar muscular atrophy also shows a specific pattern of muscle atrophy [13]. In patients with SMA, particularly type 3, the triceps brachii, quadriceps femoris, and iliopsoas are often significantly involved, while the biceps brachii and gluteus maximus are preserved [5]. In addition, the gastrocnemius tends to also be significantly involved, whereas the tibialis anterior is preserved [6]. The reason for selective muscle involvement remains unclear. D’Errico et al. hypothesized that localized vulnerability exists in the lateral funiculus of the spinal cord [14].

A report by Durmus et al. included two patients with SMA type 3 that had a long disease duration [5]. The patients, who were in their 40s, had been suffering from the disease for approximately 30 years, and remarkably, their selective muscle involvement patterns remained unchanged, despite their age and disease duration. To the best of our knowledge, this report presents the most aged case with the longest disease duration in which selective muscle involvement patterns were confirmed. This finding may suggest the concept that skeletal muscle CT scans can play a role in diagnosing advanced SMA cases. We utilized muscle computed tomography (CT) as a diagnostic tool. In previous studies, muscle magnetic resonance imaging (MRI) was often used [5,6], and other studies have shown the utility and convenience of muscle echography [15]. Muscle CT scans can evaluate the complete skeletal muscle structure of the trunk and extremities in less time than an MRI or muscle echogram. Therefore, muscle CT is suitable for assessing patients with various muscle atrophies or weaknesses, despite potential exposure to radiation.

We acknowledge that this report is limited to a single elderly patient with SMA and that younger patients with a shorter disease duration may not exhibit selective muscle involvement. Further research on SMA and other neuromuscular diseases is needed to evaluate the usefulness of muscle CT in diagnosing SMA.

## Conclusions

We described the case of an elderly patient who remained underdiagnosed for 50 years. The patient's selective muscle involvement finally led to a genetic diagnosis of SMA. Notably, this patient is the oldest known case with the longest disease duration in which selective muscle involvement patterns have been confirmed. Selective muscle involvement can be challenging to detect through physical examination, especially in the presence of other complications such as obesity, and skeletal CT scans can be helpful in those cases. Clinicians should perform genetic testing when muscle atrophy patterns are consistent with SMA to ensure a timely and accurate diagnosis.
